# Supplementary material for: Monomer and Oligomer Transition of Zinc Phthalocyanine Is Key for Photobleaching in Photodynamic Therapy
Source: Molecules. 2023 Jun 8;28(12):4639. doi: 10.3390/molecules28124639 (PMC10305241; doi:10.3390/molecules28124639)
Supplement: Supplementary file 1 [file molecules-28-04639-s001.zip › molecules-2411188-supplementary.pdf]

molecules

Supplementary Materials

Monomer and Oligomer Transition of Zinc Phthalocyanine Is Key for Photobleaching in Photodynamic Therapy

Dafeng Liu [1,2], Longguang Jiang [2], Jincan Chen [3], Zhuo Chen [3], Cai Yuan [2], Donghai Lin [1] and Mingdong Huang [2,*]

[1] MOE Key Laboratory of Spectrochemical Analysis & Instrumentation, Key Laboratory of Chemical Biology of Fujian Province, College of Chemistry and Chemical Engineering, Xiamen University, Xiamen 361005, China; dhlin@xmu.edu.cn (Donghai Lin)
[2] College of Chemistry, Fuzhou University, Fuzhou 350002, China; cyuan@fzu.edu.cn (C.Y.)
[3] State Key Laboratory of Structural Chemistry, Fujian Institute of Research on the Structure of Matter, Chinese Academy of Sciences, Fuzhou 350002, China
* Correspondence: hmd_lab@fzu.edu.cn

Table S1. Primers used in this study for oxidative stress assay.

Primer	Sequence (5′- 3′)
sodA-F<br>sodA-R	CCG ATT ATG GGC CTG GAT<br>CAA AAC GTG CCG CTG C
oxyR-F<br>oxyR-R	CGC GAT CAG GCA ATG G<br>CAG CGC TGG CAG TAA AGT G
recA-F<br>recA-R	CAT CTC TAC CGG TTC GCT TTC<br>GCG TGT TCA GCA TCG ATA AAC
LexA-F<br>LexA-R	CGG TCA GGT CGT TGT CG<br>CTG ACG AAG GTC AAC G
gyrA-F<br>gyrA-R	GTT CCA TCA GCC CTT CAA TG<br>TTG ATA ACT ATG ACG GCA CGG
espA-F<br>espA-R	GCAAACAGTGAAGGCGG<br>CACATCAGAACGTGCACTCG

Table S2. Primers used in this study for cell wall and membrane assay.

Primer	Sequence (5′- 3′)
srtA-F<br>srtA-R	GCAGCATATTTGTTTGCTAAACC<br>CAATGACACGTCGTCATTGG
murZ-F<br>murZ-R	AAAATAAGAGGTGGACGCACA<br>ACTGTTTTTCGCGCCACT
mprF-F<br>mprF-R	TCATTATTGCTGCATTATCTGGA<br>TTTTCCTCAGGGACACCTAAAG
gyrB-F<br>gyrB-R	TTAGTGTGGGAAATTGTCGATAAT<br>AGTCTTGTGACAATGCGTTTACA

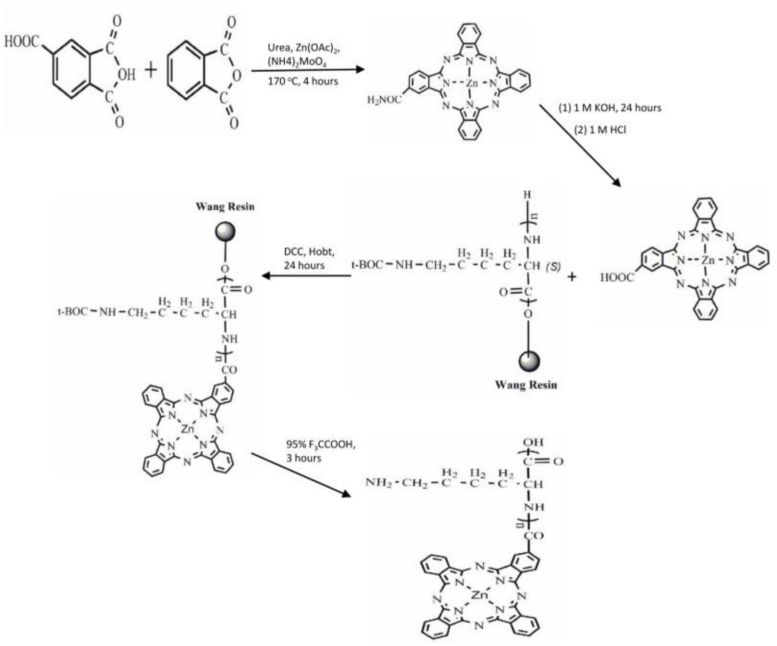

Figure S1. Synthetic route and chemical structures of ZnPc(Lys)5.

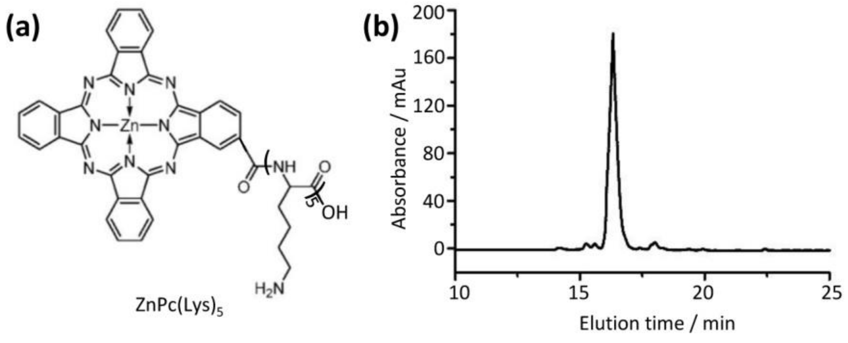

Figure S2. Characterization of PS monomer.

(a) Molecular structure of ZnPc(Lys)5. (b) ZnPc(Lys)5 was synthesized and purified to >95% purity based on HPLC profiles on C_{18} reversed-phase column, eluted by 100% MeOH/TFA.

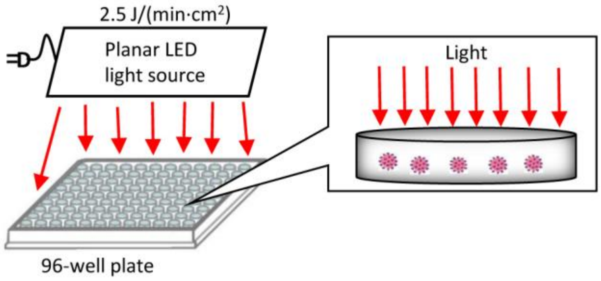

Figure S3. A planar LED light source.

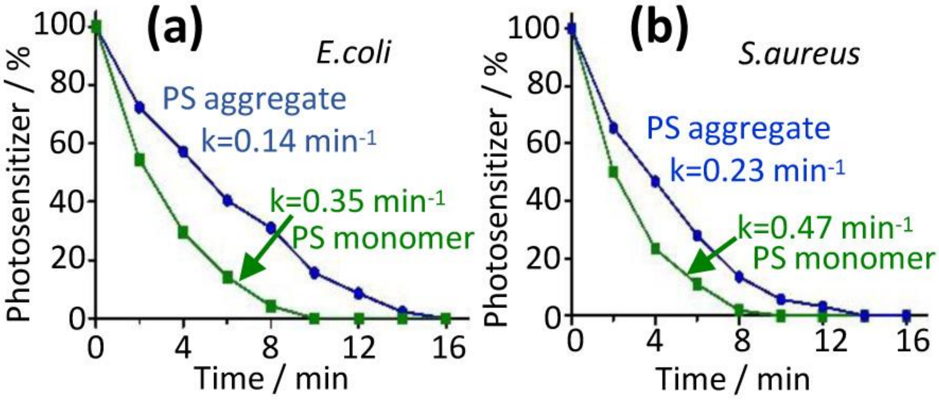

Figure S4. PS aggregate were degraded during aPDT with LED light illumination.

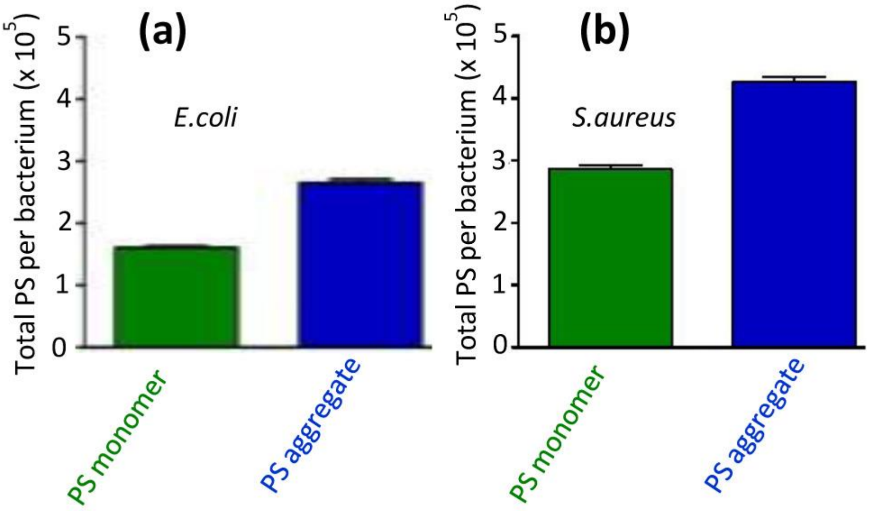

Figure S5. Amounts of PS monomer bound to bacteria was significantly less compared to PS aggregate.

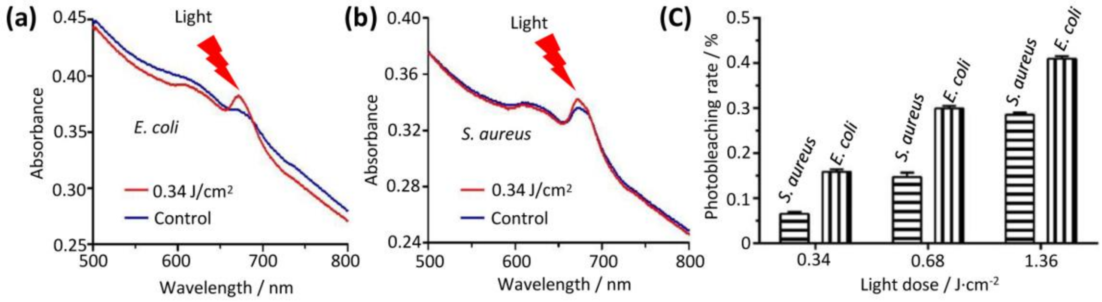

Figure S6. Adsorption intensity spectra of PS aggregation on *E. coli* (a) and *S. aureus* (b) surface was stronger at 678 nm under illumination compared to control, demonstrating that LED light facilitated disintegration of bound PS aggregation. (c) Photobleaching rates of PS monomer in microbial cell suspensions increased upon increasing light doses.

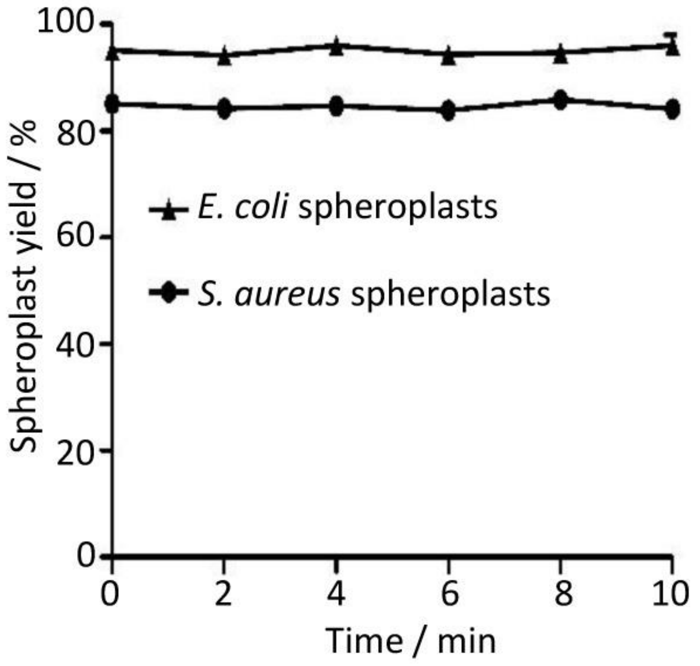

Figure S7. The yield of bacterial spheroplasts. Spheroplast yield of *S. aureus* or *E. coli* did not obviously differ with incubation time.

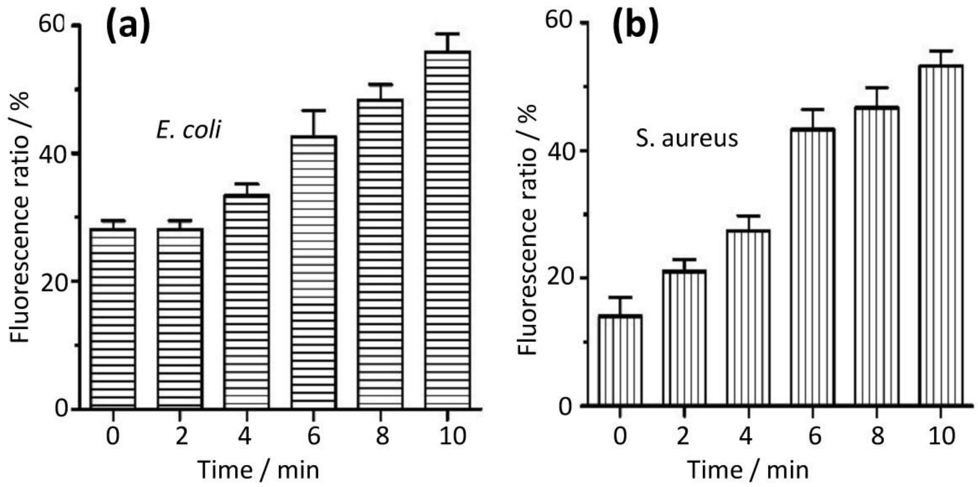

Figure S8. PS aggregation respectively was added to spheroplast suspension of (a) *E. coli* and (b) *S. aureus* in the 4th and 6th minute after spheroplasts were prepared. Fluorescence intensities of PS aggregation were measured at l_{exc}=610nm and l_{em}=680nm after bound PS aggregation to bacteria was dissolved in 0.1 M NaOH/1% SDS for 60 min.

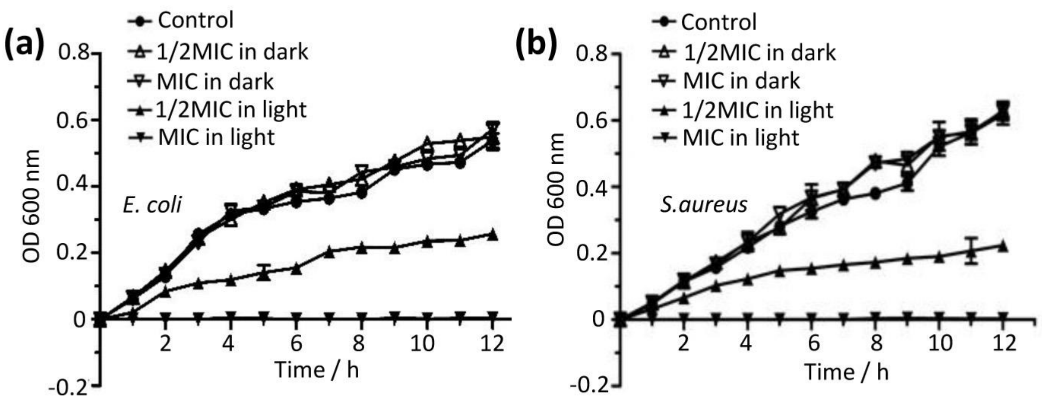

Figure S9. Growth curves of (a) *E. coli* and (b) *S. aureus* by inhibited different PS aggregation concentrations in light (light dose=12.7 J/cm^2) or dark. The PS aggregation had no toxicity to bacterial cells. In these two experiments, MICs of PS aggregation against (a) *E. coli* and (b) *S. aureus* were 1.6 and 0.8 μM, respectively.

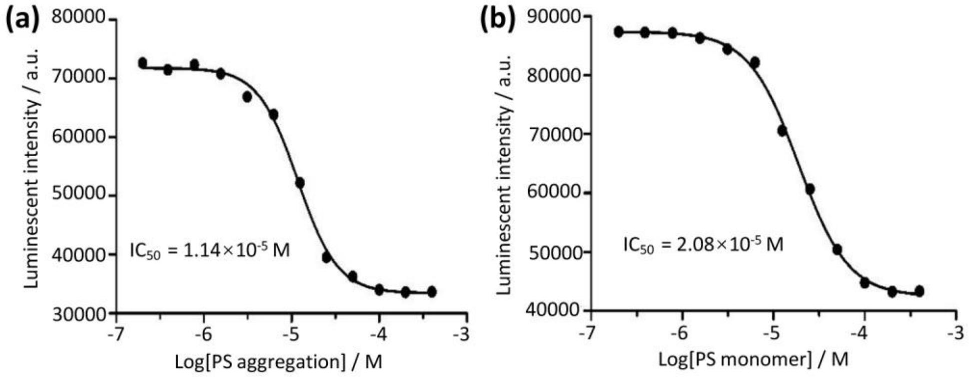

Figure S10. Dark toxicity of PS aggregation to bacteria. (a) PS aggregation and (b) monomeric PS was added into suspensions of bioluminescent *E.coli* , was incubated with bacteria in dark. The luminescent intensity was measured.

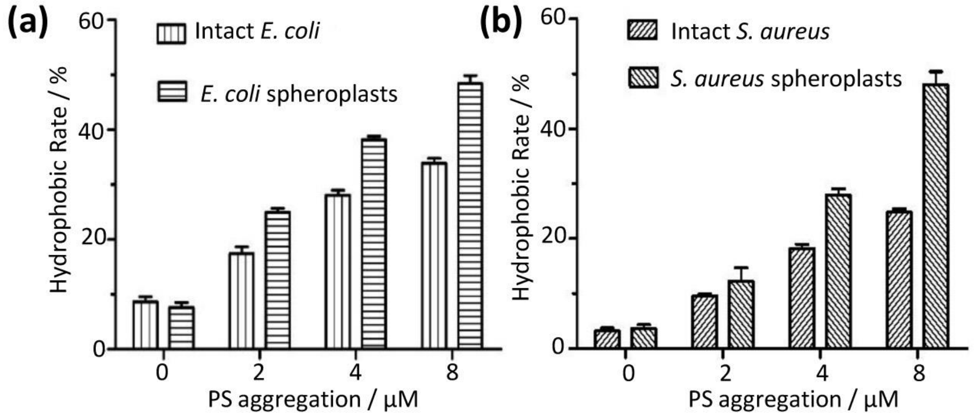

Figure S11. Hydrophobicity of (a) *E. coli* and (b) *S. aureus* increased upon increasing PS aggregation concentrations. Hydrophobicity on spheroplast surface increased faster compared to intact bacteria.

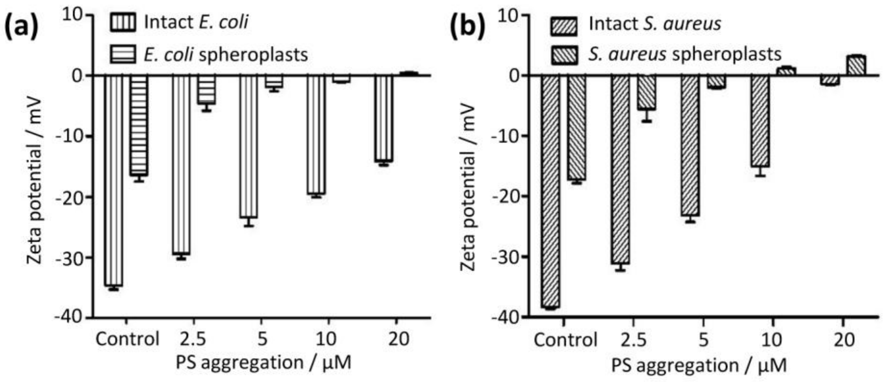

Figure S12. The PS aggregation increased zeta potential of the surface of (a) *E. coli* and (b) *S. aureus*. The PS aggregation was incubated with bacteria, and unbound PS aggregation was washed away, and zeta potential was measured.
